# Characterization of the chloroplast genome of *Zingiber mioga* (Thunb.) Rosc

**DOI:** 10.1080/23802359.2020.1866466

**Published:** 2021-02-04

**Authors:** Yeminzi Miao, Limin Chen, Hanmei Li, Dayun Zhou, Yiming Pan, Tingfu Liu

**Affiliations:** a Integrated Plant Protection Center, Lishui Academy of Agricultural and Forestry Sciences, Lishui, Zhejiang, China; b Fujian Province Key Laboratory of Insect Ecology, College of Plant Protection, Fujian Agriculture and Forestry University, Fuzhou, Fujian, China; c Lishui Vocational & Technical College, Lishui, Zhejiang, China

**Keywords:** *Zingiber mioga*, chloroplast, evolution, conservation, breeding

## Abstract

*Zingiber mioga* (Thunb.) Rosc. is an important plant species in tropical Asia widely used for decoration, and in traditional food and medicine. In this study, we reported for the first time the complete chloroplast genome sequence of *Z. mioga.* The assembled chloroplast genome was 159,868 bp long with a typical quadripartite structure consisting of two reverse repeated regions of 26,652 bp in length, separated by a large single-copy (89,431 bp) and a small single-copy (17,133 bp) region. We annotated 113 genes including 78 protein-coding, 31 tRNA and 4 rRNA genes. Phylogenetic analysis with 27 other species showed that *Z. mioga* clustered with *Z. officinale* and *Z. spectabile*, all belonging to the Zingiberaceae family of the Zingiberales order. The results of this study will facilitate the breeding process and conservation of the species.

Plant species of the genus *Zingiber* (Zingiberaceae family) are renowned for their physiological and pharmacological properties (Sharifi-Rad et al. 2017). Among them, *Zingiber mioga* (Thunb.) Rosc. is widely used as a traditional food in Asia, as an ingredient in traditional medicine (Cole and Nürnberger 2014), and as an ornamental plant. In China, *Z. mioga* is mainly exploited as wild; however, its artificial cultivation is still in early stages. Therefore, more efforts are needed toward the conservation of the species and for accelerating the breeding process. Chloroplast genome sequence provides crucial molecular tools for facilitating plant diversity studies and breeding (Daniell et al. 2016).

In this report, we sequenced and assembled the complete chloroplast genome of *Z. mioga* (GenBank accession number: MW067010) and performed a phylogenetic analysis with 27 other species.

Fresh leave samples of *Z. mioga* were collected in wild from a forest (N: 28.412585″, E: 119.355606″), near Daitou Village, Yecun Town, Songyang County, Zhejiang Province, China, 1080 m altitude, on 14 April 2020. The genomic DNA was extracted from fresh leaves using the cetyltrimethylammonium bromide (CTAB) protocol (Doyle and Doyle 1987). The voucher specimen was deposited at Lishui Academy of Agricultural and Forestry Sciences (Accession number: SY1). The sequencing library was prepared according to the standard protocol provided by Illumina (Illumina, San Diego, CA) using the NEBNext Ultra™ DNA Library Prep Kit. The chloroplast genome was paired-end (150 bp) sequenced at Wuhan bio-mall biotechnology Co., Ltd, Wuhan, China, using the Illumina NovaSeq platform (Illumina, San Diego, CA).

A total of 3.956 Gb of raw reads were generated, and by employing the tool SOAPnuke (v1.3.0) (Chen et al. 2018), the reads having adapter contamination and those with more than 5% unknown bases were removed to obtain clean reads. Finally, 99.8% of the raw reads were declared as clean reads for downstream analyses. The chloroplast genome was reconstructed using SPAdes (v3.13.0; parameter: -k 127) (Nurk et al. 2013), and Gapcloser (v1.12) was further employed to fill the gaps. The final assembly resulted in a total length of 159,868 bp, with a GC content of 36.36% and an assembly coverage of 660X. The chloroplast genome had a typical quadripartite structure consisting of two reverse repeated regions of 26,652 bp in length, separated by a large single-copy (89,431 bp) and a small single-copy (17,133 bp) region.

The chloroplast genome functional annotation includes encoded gene prediction and non-coding RNA annotation (rRNA and tRNA). We used the GeSeq software (Tillich et al. 2017), and BLASTn (BLAST 2.2.30 +; parameter: -evalue 1e–5) (Altschul et al. 1990) for the chloroplast genome annotation. In total, 113 genes were identified including 78 protein-coding , 31 tRNA , and 4 rRNA genes.

In order to clarify the phylogenetic position of *Z. mioga*, the chloroplast genome sequences of 27 species from 10 different families (Zingiberaceae, Costaceae, Cannaceae, Marantaceae, Lowiaceae, Strelitziaceae, Musaceae, Dioscoreaceae, Hanguanaceae, Bromeliaceae) were retrieved from GenBank of National Center for Biotechnology Information (NCBI at https://www.ncbi.nlm.nih.gov) (accession numbers provided in Figure 1). The complete chloroplast genome sequences were aligned using MAFFT version 7.0 (Katoh and Standley 2013), and a Neighbor-Joining phylogenetic tree was constructed with 1000 bootstraps using the program Treebest (v1.9.2; parameter: nj). Our result showed that *Z. mioga* clustered together with *Z. officinale* and *Z. spectabile* (Figure 1), all belonging to the Zingiberaceae family of the Zingiberales order.

**Figure 1.**
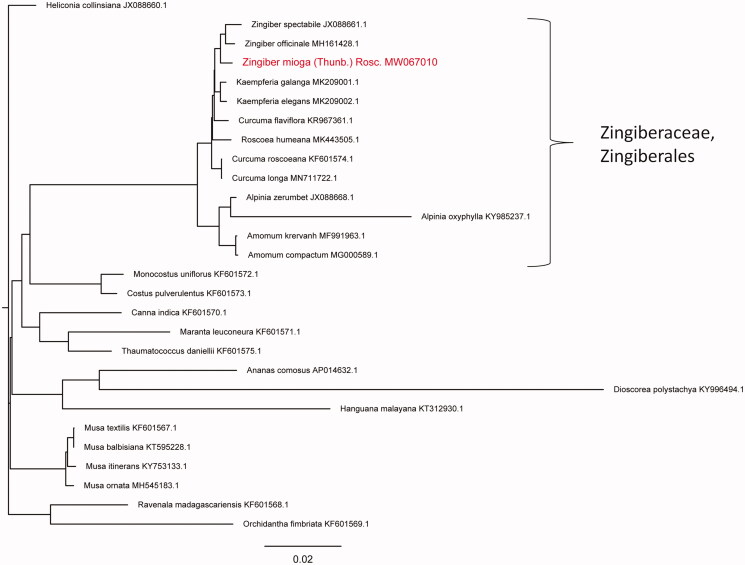
Neighbor-joining phylogenetic tree based on the chloroplast genome sequences from 28 species. Values along branches correspond to bootstrap percentages.

## Data Availability

The genome sequence data that support the findings of this study are openly available in GenBank of NCBI at (https://www.ncbi.nlm.nih.gov/) under the accession no. MW067010 (https://www.ncbi.nlm.nih.gov/nuccore/MW067010). The associated BioProject, SRA, and Bio-Sample numbers are PRJNA681416, SRX9603488, and SAMN16953478, respectively (https://www.ncbi.nlm.nih.gov/bioproject/?term=PRJNA681416).
